# Timely Digital Patient-Clinician Communication in Specialist Clinical Services for Young People: A Mixed-Methods Study (The LYNC Study)

**DOI:** 10.2196/jmir.7154

**Published:** 2017-04-10

**Authors:** Frances Griffiths, Carol Bryce, Jonathan Cave, Melina Dritsaki, Joseph Fraser, Kathryn Hamilton, Caroline Huxley, Agnieszka Ignatowicz, Sung Wook Kim, Peter K Kimani, Jason Madan, Anne-Marie Slowther, Mark Sujan, Jackie Sturt

**Affiliations:** ^1^ Division of Health Sciences Warwick Medical School University of Warwick Coventry United Kingdom; ^2^ Centre for Health Policy School of Public Health University of the Witwatersrand Johannesburg South Africa; ^3^ Department of Economics University of Warwick Coventry United Kingdom; ^4^ Oxford Clinical Trials Research Unit Nuffield Department of Orthopaedics, Rheumatology and Musculoskeletal Sciences University of Oxford Oxford United Kingdom; ^5^ Patient and Public Involvement Coventry United Kingdom; ^6^ Florence Nightingale Faculty of Nursing and Midwidery King's College London London United Kingdom

**Keywords:** digital communication, long-term conditions, young people, digital health care, patient communication, NHS, National Health Service

## Abstract

**Background:**

Young people (aged 16-24 years) with long-term health conditions can disengage from health services, resulting in poor health outcomes, but clinicians in the UK National Health Service (NHS) are using digital communication to try to improve engagement. Evidence of effectiveness of this digital communication is equivocal. There are gaps in evidence as to how it might work, its cost, and ethical and safety issues.

**Objective:**

Our objective was to understand how the use of digital communication between young people with long-term conditions and their NHS specialist clinicians changes engagement of the young people with their health care; and to identify costs and necessary safeguards.

**Methods:**

We conducted mixed-methods case studies of 20 NHS specialist clinical teams from across England and Wales and their practice providing care for 13 different long-term physical or mental health conditions. We observed 79 clinical team members and interviewed 165 young people aged 16-24 years with a long-term health condition recruited via case study clinical teams, 173 clinical team members, and 16 information governance specialists from study NHS Trusts. We conducted a thematic analysis of how digital communication works, and analyzed ethics, safety and governance, and annual direct costs.

**Results:**

Young people and their clinical teams variously used mobile phone calls, text messages, email, and voice over Internet protocol. Length of clinician use of digital communication varied from 1 to 13 years in 17 case studies, and was being considered in 3. Digital communication enables timely access for young people to the right clinician at the time when it can make a difference to how they manage their health condition. This is valued as an addition to traditional clinic appointments and can engage those otherwise disengaged, particularly at times of change for young people. It can enhance patient autonomy, empowerment and activation. It challenges the nature and boundaries of therapeutic relationships but can improve trust. The clinical teams studied had not themselves formally evaluated the impact of their intervention. Staff time is the main cost driver, but offsetting savings are likely elsewhere in the health service. Risks include increased dependence on clinicians, inadvertent disclosure of confidential information, and communication failures, which are mostly mitigated by young people and clinicians using common-sense approaches.

**Conclusions:**

As NHS policy prompts more widespread use of digital communication to improve the health care experience, our findings suggest that benefit is most likely, and harms are mitigated, when digital communication is used with patients who already have a relationship of trust with the clinical team, and where there is identifiable need for patients to have flexible access, such as when transitioning between services, treatments, or lived context. Clinical teams need a proactive approach to ethics, governance, and patient safety.

## Introduction

Young people living with long-term conditions are vulnerable to disengagement from health care, which endangers their current and future health [1-5]. Health service factors affecting young peoples’ engagement with health care include poor patient-clinician communication, inflexible access to people and information, lack of person-centered health care, and the need for continuity and relationship development [2,6-9].

In the United Kingdom, 90% of young people aged 16-24 years own a smartphone [10]. Studies have reported requests from young people to be able to communicate via email, text message, and social media with their health care team [6,11]. There are reports of specialist clinical teams using digital channels for monitoring and information sharing [12,13]. In the United Kingdom, government policy and investment is driving the digitization of the National Health Service (NHS) [14,15]. With the rollout of NHSmail 2 [16], NHS clinicians now have access to secure email and other digital channels for communicating with patients on clinical matters.

Evidence for effectiveness on health outcome of the use of digital channels with patients on clinical matters is not strong. Prior to starting our project, we found 16 systematic reviews [17-32] and 1 clinical review [33] published from 2010 to 2012 on the effectiveness of digital communication between clinicians and patients with long-term conditions, where the long-term condition was relevant to young people (only 2 reviews focused on young people [25,32]). Evidence of an impact on clinical outcomes was equivocal, although no trials reported poorer outcomes in the intervention arm. The reporting of interventions was generally poor. The systematic reviews identified the following gaps in evidence: how digital communication might work [19,21,27,30,33,34], in particular examining the function of the communication rather than the communication channel [33]; what was important to patients and clinicians [19,20,22,23,25-27,32,33,35]; cost and resource use [17,19,21,23,25,27-29]; risks including privacy and data security [19,22,23,27,33]; the need to focus on widely used digital communication rather than being future focused [29]; and research to inform policy, practice, and implementation or rollout [21,22,28].

Given the poor quality of the evidence, and the gaps in the evidence about how digital communication might work, its value to patients and clinicians, and its cost and risks, we had the following aims. First, we wanted to identify how the use of digital channels for communication between young people and their clinicians was addressing the health service factors influencing young people’s engagement with health care, and the perceived impact and value of the digital communication. Second, we aimed to identify cost, ethical, and patient safety issues that need to be considered in the NHS policy-driven rollout of digital communication. To meet both these aims, we studied NHS clinicians and young people with long-term conditions requiring specialist care, who were already using, or considering using, digital channels for communication about clinical issues, where the communication was two-way (synchronous or asynchronous), and where both the clinician and the young person could be mobile.

## Methods

This was an observational mixed-methods study of cases [36] undertaken in the UK NHS, where services are free at the point of delivery.

### Case Study Sampling

We used multiple strategies to identify clinical teams. First, between December 2013 and February 2014, using Google (Google Inc, Mountain View, CA, USA), we searched the Internet for reports of the use of digital communication with patients in the NHS using the keywords “e-health,” “telehealth,” “telemedicine,” “digital communication,” “young people,” and “young persons.” We scrutinized the first 35 pages of each search for relevant reports. Further information was then sourced from individual NHS Trust websites, documents, and reports and by contacting key individuals. Second, we listed the project on the UK National Institute for Health Research (NIHR) portfolio inviting participation. Third, we contacted clinicians we knew personally or had encountered at applied health conferences and asked them to distribute information about the project to their networks. Fourth, clinical teams expressing interest in the study were asked to pass on the study information to potentially interested colleagues.

Study inclusion criteria were that (1) the clinical team was providing specialist care for young people (age 16-24 years) with long-term conditions (eg, sickle cell, liver disease, cystic fibrosis, cancer, or mental health issues), (2) the team had interest in the use of two-way digital communications with the young people, and (3) the long-term condition had considerable cost implications for the NHS.

We sampled 20 teams purposively for diversity of clinical condition, use of digital communication with patients, size, and geographic location. Studying 20 teams ensured both diversity and anonymity of study teams. Anonymity was important as; at the time of undertaking the study, some study teams may have been in breach of information governance policies.

We obtained ethical approval (14/WM/0066) from National Research Ethics Service Committee West Midlands - The Black Country.

### Data Collection

We collected data between November 2014 and March 2016. Prior to commencing fieldwork with each clinical team, we requested to see any in-house evaluations of their digital communication with patients that they had done. During recruitment of clinical teams, some teams mentioned that they were using digital channels without formal approval from their Trust. We therefore sought to interview the Trust information governance specialist before collecting data from any clinical teams.

To understand how digital clinical communication was used, including its perceived impact, and to identify issues related to ethics and patient safety, we observed and interviewed clinical team members at all study sites. We collected data within a 2-week data collection period during the team’s day-to-day work, recruiting as many team members as were prepared to participate. To explore the cost of using digital communication with patients, we collected data on equipment and clinician time spent on the use of digital communication with patients and its cost. To gather these data, we developed a questionnaire based on early interview data and used this as part of clinical team interviews.

We also recruited for interview young people aged 16-24 years under the care of the clinical team and due to be seen by the team during the 2-week data collection period. The young people were sent study information prior to their scheduled encounter. The clinical team or the study researcher approached each young person at the scheduled encounter—usually before the appointment time—to take consent and confirm interview arrangements. Those not attending were further contacted to request participation. At the interview, we asked the young people about their use of digital communication with the clinical team, its impact on their day-to-day life and ability to manage their health condition, and ethical and safety issues. To explore the value young people placed on digital access to their clinical team, we asked them what they would be willing to pay for the service. Young people were each offered a £20 store voucher as a thank-you token. Interviews used any communication channel preferred by the participant, such as phone, in person, or email. We recruited for interview until we were confident we were not gaining any new data from the young people on their experience and views of the use of digital communication with clinicians at their clinic.

### Data Management and Analysis

Observation notes were taken, and then typed up and expanded immediately after observation. Interviews were audio-recorded or notes taken, typed up, and expanded. We made reflective notes after each observation or interview. Recordings were transcribed and checked for accuracy. All identifiers were removed, and data were identified with a site and participant number. Independent coding was undertaken on 20% of all coding, and discrepancies were discussed. Quality checks were undertaken on data entry of survey data.

We coded all qualitative data for the major prespecified analysis themes related to our aims: (1) how digital communication with patients is used and its perceived impact, (2) the value of the communication to young people, (3) its ethical impact, and (4) patient safety and governance issues and their mitigation. Within these coded data, we undertook (1) further analysis identifying the mechanisms by which the digital communication had an immediate impact, and its context [37], (2) thematic analysis [38], (3) thematic analysis informed by theory [39], and (4) thematic analysis with an established safety framework [40].

Using staff questionnaire data, we calculated, for each respondent, the annual direct costs associated with digital communication with patients. We used NHS Agenda for Change pay scales 2014-2015 [41] for salaries and University of Warwick information technology service price lists for employer-provided equipment, annualized assuming a 3-year life span and a discount rate of 3.5% [42]. We estimated total costs at each site where over 50% of clinical team members responded to the survey. Where data permitted, we estimated cost per patient based on the size of each clinic’s patient list.

### Patient, Public, and Stakeholder Involvement

We explored early research ideas with an experienced Patient, Public, and Stakeholder Involvement group with which we had worked for over 10 years (Warwick Diabetes Research and Education User Group). Subsequently, to gain input from young people, 20 students (15-17 years of age) from 5 local schools collected opinions from their peers and reported this as a film [43]. Patient, Public, and Stakeholder Involvement coapplicant and coauthor JF drew on this to advise the project team about collecting data from the young people living with long-term conditions. He subsequently chaired the project management group. This group included 4 young adults and a parent of a young person living with a long-term condition, and representation from NHS Digital (UK Department of Health, Leeds, UK). They advised on recruitment, data collection procedures, analysis, and impact strategies.

Each clinical team is receiving a copy of the project report. Study results in the form of Quick Reference Guides [44] are being disseminated to patient support and advocacy groups, professional organizations, and all NHS Trusts, Health Boards, and Clinical Commissioning Groups.

## Results

### Study Sites and Participants

**Figure 1:**
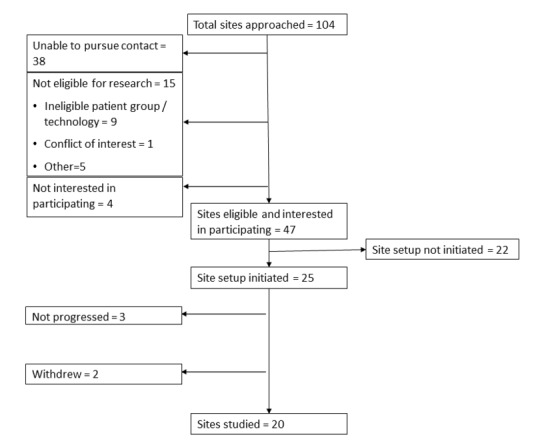
Flowchart showing case site recruitment.

We identified 104 clinical teams (via Internet search, 15; NIHR portfolio, 7; networks and contacts, 58; contacts of already interested clinical teams, 24), of which 47 were eligible and interested in participating (see Figure 1). We initiated site setup at 25 sites and studied 20, covering 13 clinical specialties (see Table 1). Clinic populations included children and adolescent services, transition services, young adult services, and adult services. There were 9 clinical teams in the South and East of England, 7 in the Midlands, 3 in the North of England, and 1 in Wales. At recruitment, clinical teams variously reported using with their young people the following communication channels: mobile phone calls, text messages, email, voice over Internet protocol, and personal health records. A total of 3 clinical teams used no digital communication with their young people.

**Table 1:** Case study site health condition, clinic type, age group, digital communication used with patients, and data collected.

Site identifier	Clinic population^a^	Patient age range (years)	Digital communication channels used in clinic	No. of young people interviewed	No. of staff interviewed	No. of information governance specialists interviewed	No. of staff shadowed
Diabetes 1	Transition	12-19	Mobile phone, text message, email	12	8	0	5
Mental Health 1 (Early Intervention)	Age independent	>16	Mobile phone, text message, email	1	8	1	2
Cystic Fibrosis 1	Adult	>16	Email	2	5	1	1
Dermatology	Adult	>18	Email	7	4	0	2
Mental Health 2 (CAMHS^b^)	Child and adolescent	<18	None	4	11	1^c^	4
Mental Health 3 (Outreach team)	Child and adolescent	<18	Mobile phone, text message, VoIP^d^	5	11	1	3
Arthritis	Transition	16-25	None	16	8	1	1
Cystic Fibrosis 2	Adult	>16	Mobile phone, text message, VoIP	13	11	0	3
School nurse service	Young people	14-19	Text message, VoIP (pilot)	0	7	1	4
Kidney	Young adult	16-22	Email	7	7	0	3
Liver	Transition	12-25	Text message, email	15	12	2	7
Sickle Cell	Transition	12-24	Mobile phone, text message	10	13	2	9
Mental Health 4 (Early Intervention in Psychosis Team)	Youth	14-35	Mobile phone, text message, email	4	9	2	8
Cancer 1	Teenage and young adult	15-24	Mobile phone, text message, email	12	7	0	3
Diabetes 2	Transition	16-25	Mobile phone, VoIP	11	6	2	2
Inflammatory Bowel Disease 1	Adult	>16	Web portal, email	1	6	1	3
Inflammatory Bowel Disease 2	Adolescent	13-23	Email	13	7	0	4
HIV^e^	Adult	>18	None	9	12	0	4
Sexual Health	Adult and young people	>16	Testing kits ordered online	12	10	0	3
Cancer 2	Teenage and young adult	15-24	Mobile phone, text message, email	11	11	2	8

^a^As described by clinic staff.

^b^CAMHS: Child and Adolescent Mental Health Services.

^c^Information governance specialist was the same person as for Mental Health 1.

^d^VoIP: voice over Internet protocol.

^e^HIV: human immunodeficiency virus.

We recruited for interview 165 young people. Interviews were undertaken by phone (n=82), face-to-face (n=41), email (n=35), Facebook (n=4), Skype (n=2), and text message (n=1). Speech-based interviews lasted 20-60 minutes, with the majority lasting approximately 30 minutes. Text message-based interviews took up to 2 weeks.

We recruited 16 information governance specialists and 173 clinical team members for interview. The clinicians included consultants, registrars, community nurses, advanced nurse practitioners, psychologists, dietitians, physiotherapists, occupational therapists, and pharmacists (7 interviews were with clinic administrators closely involved with patient care). Interviews were undertaken face-to-face (n=158) or by phone (n=31) and lasted up to 2 hours, with the majority lasting approximately 45 minutes. We shadowed 79 clinical team members, usually for 1-2 hours, longer if appropriate (eg, when observing home visits with a clinician). Of the 173 clinical team members, 115 completed staff health economic questionnaires across 18 sites.

### Timely Digital Contact Between Young People and Their Clinical Team

Young people and clinicians mostly used digital channels to be in contact at times when the contact could make a difference to how the young people managed their condition. This timely access was not scheduled, although clinicians often planned their contacts.

The channels of communication used for this timely access varied across clinical teams (see Table 1) and according to the reason for making contact. Mobile phones were used for urgent issues and when discussion was needed to resolve the clinical problem. Text messaging was used for keeping in touch, raising less-urgent concerns such as new symptoms or changing trends in home monitoring (eg, blood sugars), personal reminders about upcoming appointments, and reminders about therapy. Additional clinical team members used text messaging to make direct contact with young people where parents were involved in a young person’s treatment, as this allowed issues to be raised that would not be raised in front of parents. Email was useful for sending complex information and summaries of discussion at a consultation, as the young people were then able to read and reread the information, and for sending test results where the results were routine or as expected and the individual was well known to the service. The young people emailed questions such as how to use a skin cream or fix equipment, concerns such as suitability of vaccinations for travel, photographs of their condition, such as a rash, and requests for supplies.

Although the clinical teams we studied were motivated to use timely digital communication with their young people to improve their health outcome, none of the clinical teams had evaluated the impact of its use on health outcome. However, our data revealed many mechanisms by which timely digital access improved health care and so had the potential to improve health outcome. Young people and clinicians reported that timely digital communication enhanced engagement, reduced patient anxiety, and improved trust between the young people and their clinicians. Young people felt they received personalized care and valued the continuity of care they received by being able to contact the clinicians who knew them when they needed to. The timely access prompted activation and better self-management by the young people:

I sort of just avoided doing anything really and just thought it might sort itself. But...I do need to accept the help that’s out there for me...it’s a lot better just being able to speak over email and then when you do need a test done you’re only going into your doctors every four or five months, if that.

Young person 06, Diabetes 2

I can email them anytime, I can get a response anytime and sort it out myself.

Young person 07, Inflammatory Bowel Disease 2

Young people who were already engaged with their clinical team sometimes used email or text messaging to communicate about sensitive issues that they found difficult to raise face-to-face, knowing that the email or text message would prompt the clinician to raise the issue when they saw them. Some disengaged young people reengaged with their clinical team via email or text message when the young person had not responded to phone calls or regular mail.

Young people and clinicians reported examples of where timely digital communication had been used to diagnose minor issues, sometimes avoiding unnecessary clinic visits, or to treat symptoms before they became serious, thus avoiding the need for emergency care.

Timely digital access was considered, by both young people and their clinicians, to be a valuable addition to traditional clinic appointments, not a replacement. Face-to-face communication was considered important for establishing relationships and for conveying bad or potentially upsetting news. The use of digital channels for routine issues and exchange of information between appointments left more time in clinic appointments for complex issues, and so increased the value of the face-to-face consultations for both the young people and clinicians.

Digital communication was convenient for the clinicians and young people, as it avoided disruption to their other activities and sometimes avoided unnecessary consultations. The use of asynchronous communication such as email and text messaging allowed them to think about their questions or responses. Clinicians liked the opportunity to consult clinical records before responding, although where a clinician knew the patient well they did not always do this.

### Value to the Young People and Cost to the NHS of Timely Digital Access

A total of 110 of the 165 young people answered the exploratory question on their willingness to pay for digital communication with their clinical team. The median willingness to pay was £5 per month (interquartile range £0-£16, maximum £120). A total of 27 young people reported being willing to pay £30 or more per month, 35 were willing to pay between £0 and £30, 30 would not be willing to pay extra, and 18 were unable or unwilling to answer this question. The reasons young people gave for wanting the service mostly related to resolving problems quickly, such as an issue with self-injection; enabling easier contact with a named clinician for continuity of care; and saving time travelling to a clinic to report progress and to hear or provide results.

Young people from one mental health site objected to the question on the basis that the service should be free, or because of a perception that payment would be discriminatory if applied only in their service. Those not willing to pay were not currently using digital channels for communicating with their clinical team, even where they were available, and thought that conventional channels could be used just as well.

**Figure 2:**
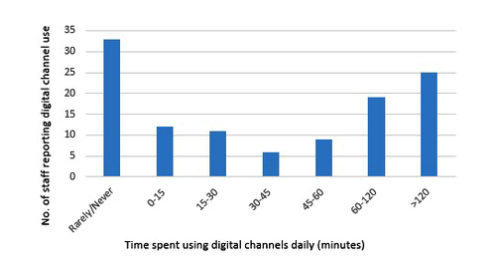
Minutes per day reported by clinical team members (n=115) as spent using digital communication with patients.

Our exploratory data indicated that the mean time spent by staff per day using digital channels to communicate with young people was 76 minutes (median 45 minutes, interquartile range 0-120 minutes). The mean and median times were not typical levels of activity (see Figure 2): 33 of 115 staff (28.7%) reported using digital communication with patients “rarely” or “never,” and 25 staff (21.7%) reported using it for over 2 hours per day. Use varied by grade and profession. Medical consultants reported substantially lower use (mean time 28 minutes per day) than nurses (120 minutes per day) and physiotherapists (120 minutes per day), but consultants’ use was similar to that of dietitians (14 minutes per day) and psychologists (34 minutes per day). The major cost for providing digital communication between clinic appointments was staff time (see Table 2). Staff time was typically 90%-95% of total cost. For sites where the clinic was able to provide the size of their patient list so that cost per patient could be calculated, the reported figure was between £0 and £20 per month, with the exception of the 2 cystic fibrosis sites, where costs per patient were much higher (£73-£130).

**Table 2:** Site-level costing analysis.

Site	No. of health economic questionnaires completed	Cost per month (£)			
		Clinical team cost	Equipment cost	Total cost	Average cost per patient
Cancer 1	5	2920	97	3017	N/A^a^
Mental Health 3 (Outreach team)	8	9230	330	9560	N/A
Arthritis	8	0	0	0	0
Kidney	6	135	26	161	16
Diabetes 1	6	2648	85	2733	4
Cystic Fibrosis 1	11	5323	383	5706	73
Sexual Health	10	3673	120	3793	N/A
HIV^b^	9	1055	51	1106	N/A
Cancer 2	11	6090	267	6357	N/A
Inflammatory Bowel Disease 1	7	3604	26	3630	3
Mental Health 2 (CAMHS^c^)	6	212	18	230	2
Liver	7	3806	71	3877	N/A
Inflammatory Bowel Disease 2	4	2672	63	2735	N/A
Cystic Fibrosis 2	3	1490	69	1559	130
Dermatology^d^	2	–	–	–	–
Mental Health 4 (Early Intervention in Psychosis Team)^d^	4	–	–	–	–
Sickle Cell^d^	6	–	–	–	–
Diabetes 2^d^	2	–	–	–	–

^a^N/A: not available.

^b^HIV: human immunodeficiency virus.

^c^CAMHS: Child and Adolescent Mental Health Services.

^d^Insufficient data to calculate clinic costs.

Most staff reported that their workload was manageable. When asked what they would do without digital communication, they said they would spend time trying to contact young people by phone or arranging appointments for them. However, staff did not report that digital communication reduced their workload overall, and several reported an increase.

From the qualitative data we identified mechanisms by which NHS costs may be reduced through the use of digital communication with patients. These were a follows:

- Reducing costly complications of illness through early treatment
- Reducing the number of appointments young people had to attend
- Reducing “did not attend” rates
- Responding to queries, for example, for young people with sickle cell, to avoid a visit to an emergency department
- Improving response to therapy through provision of advice and support, so reducing future health care cost.

### Managing Access Through Digital Channels

Although the ease of access that digital channels allowed was appreciated by both young people and clinicians, *both* were aware of the need to manage expectations. These were still being worked out by some clinical teams and their young people. Clarity about response times, working hours, and the channels of communication suitable for different purposes was considered important. Young people and clinicians wanted this information to be easily available through email and text messages, bounce-back messages, and voicemail and to be reinforced during consultations and communications. Clinical teams reported response times between a few minutes to a few days, depending on the health condition and channel of communication. Poor network coverage in some rural areas and the cost to young people of digital communication were identified as limiting digital access for some young people.

### Ethical Impact of the Use of Timely Digital Communication

Digital communication has the potential to both enhance and undermine patient autonomy. Clinicians explained that it increased patient autonomy [45] by giving the young people more control in both the management of their condition and the way in which they communicated with their clinical team. But they also noted that it may discourage some young people from taking responsibility for their own health by providing easy access to a decision maker. Young people placed more emphasis on the personalization of their care with digital communication than on increased empowerment. The ability to have more frequent contact with a specific clinician meant the clinician was more likely to know that particular young person, their circumstances, and what is important to them, so enabling the clinician to deliver person-centered care [46]:

Your relationship with the nurse is a lot easier...because they know you and they know your condition...[rather]than just another nurse that you come to see. They understand how yours is different to everybody else’s.

Young person 04, Diabetes 1

Communicating digitally reduced the power imbalance in the patient-clinician relationship, with clinicians fitting into the young person’s world rather than the young person being expected to fit into the clinical world. However, there were consequences for clinician autonomy. Clinicians expressed concerns about blurring of the patient-clinician boundary:

[The patient]started sending me huge numbers of emails and chasing me a lot...I had to then think about what’s a reasonable time frame for getting back to[the patient].

Consultant 04, Mental Health 3

Another concern was losing control over clinician information; one clinician reported how their patient had put the clinician’s text messages out on social media.

The concept of a duty of care to an individual patient is enshrined in professional codes and common law [42,47]. The development of a more personalized relationship through digital communication created uncertainty for both patient and clinician about their understanding of the duty of care and its limits. Clinicians described their concerns about the patient’s use of text messaging or email for communication about serious health concerns outside of the clinic’s normal working hours. They were unsure where the boundary was to the duty of care:

I was worried she [the patient] was going to do something dangerous like commit suicide or something, because she has mental health issues. And then felt awful the fact that I’d given her my email as a point of contact and then she’d reached out but it was two o’clock in the morning and of course I hadn’t picked it up.

Clinical team member 01, Liver Disease

However, across all sites, few participants were able to recall an instance where a patient had left an urgent communication that was not picked up in a timely manner.

There is an implied promise at the heart of the patient-clinician relationship that information disclosed to the clinician by the patient, or gained in the process of that patient’s care, will not be disclosed to others without the patient’s consent. Young people varied in their level of understanding of, and concern about, confidentiality and privacy. Clinicians were usually cautious about sending confidential data digitally, and many distinguished between a clinician sending information to the young person (risk of breach of confidentiality) and the young person sending data to the clinician (young person’s choice and their responsibility).

### Patient Safety

In addition to the inadvertent disclosure of sensitive information discussed above, our data revealed three other major categories of hazards from the use of timely digital communication between young people and their clinical team: communication failures; failure to record the content of the communication; and failure to consult the patient’s notes prior to engaging in communication. Table 3 summarizes the causes, consequences, and current form of mitigation of these hazards. These hazards are common to all forms of clinical communication, but the ease and speed of use of digital channels magnifies the risks.

**Table 3:** Hazards, consequences, causes, and current form of mitigation identified by young people with long-term conditions and their clinicians using digital channels to communicate about clinical issues.

Hazard	Consequences	Causes	Current form of mitigation
Inadvertent disclosure of sensitive information	Negative effects on patient wellbeing; jeopardizing trust between clinician and patient	Hacking, interception of communication, loss or theft of hardware, poor usability of encrypted mail service, shared email accounts and computers, sending communication to wrong recipient, excessive distribution of communication	Limiting the use of digital communication; technical solutions; double-checking contact details; ensuring explicit or implicit patient consent
Communication failures	Failure or delay in providing relevant clinical information and advice; patients discouraged from seeking relevant advice; delays in escalation to emergency care; unnecessary escalation to emergency care; patient uncertain or anxious; clinician stressed or anxious	Not answering communication from unknown numbers, not being able to establish the patient’s identity, delay in picking up or responding to messages, inability to access the Internet on mobile phones due to lack of signal or credit, poor usability of devices, difficulty expressing clearly information requests in text messages, patients downplaying seriousness of their condition in text messages, difficulty of checking correct understanding of communication content using asynchronous channels	Limiting the use of digital communication; clinician training in use of asynchronous digital channels with patients; planning for sufficient time to write and read digital communication carefully; using alternative means of emergency advice seeking; following up using a different communication channel
Failure to record content of digital communication	Other clinicians unaware of prior communication; unnecessary duplication of questions and advice given to patients; gaps in clinical record; lack of clarity for patients and clinicians about what was communicated	Digital communication not logged automatically; content of text and email messages not easily transferred to clinical notes; time consuming to record all digital communication; limited storage on communication device; lack of common understanding of how to document content of digital communication in clinical record	Treating every communication as equivalent to a face-to-face consultation; limiting the use of digital communication to forms readily integrated with patient’s clinical record; restricting the use of digital communication
Failure to consult patient’s notes prior to engaging in digital communication	Reliance on an incomplete understanding of patient’s clinical history; duplicate or contradictory advice giving	Perceived familiarity with the patient because of frequent contact; acute problem requiring urgent response; nonclinical nature of many of the digital communications between young person and clinician	Familiarity with the patient; double-checking notes after the communication has taken place

Information governance specialists expressed a willingness to support their clinical teams in using digital communication to improve health care. They are also required to monitor adherence to NHS Trust policy. Of the 16 information governance specialists interviewed, 13 reported the existence of policies in their organizations that specifically covered text messaging, emailing, and the use of handheld mobile devices. They recognized that policies need to evolve as digital communications evolve, with a majority of those interviewed currently developing policies. A few Trusts did not permit digital communication with patients, and some information governance specialists in these Trusts were aware that it was nonetheless taking place. During their interviews, information governance specialists discussed the hazards of digital communication with patients in general terms and recognized the need for training clinical teams in its use. None of the clinical teams we studied reported that they had undertaken a formal patient ethical or safety appraisal of their service. As Table 3 describes, young people and clinicians were often left to mitigate the risks by relying on common-sense strategies (eg, escalation by other means for emergencies) and by restricting the use of digital communication (eg, restricting it to nonurgent matters). A trusting relationship between the young people and their clinical team was important for mitigating both patient safety and ethical risks.

## Discussion

### Principal Findings

The provision of timely digital communication between young people with long-term conditions and their clinicians is addressing the health system factors that in the past have led to these young people disengaging from health services. Digital channels enable contact between young people and their clinical teams when this contact can make a difference to how the patient manages their condition. This digital service improves the patient’s experience of and engagement with care and prompts greater levels of self-management. Offering both digital and face-to-face contact is important to young people and clinicians. It also has the potential to reduce health care inequalities by engaging young people who are otherwise hard to reach. Young people value the enhanced access. Providing this access increases staff workload. The cost of providing this access is mostly attributable to staff costs. This cost is not immediately apparent to patients in the NHS, where services are free at the point of delivery. There is potential for offsetting savings from reduced adverse events and enhanced long-term outcomes, but these will not generally accrue to the service facing increased initial costs. As NHS policy prompts further rollout of digital access between patients and clinicians, there are ethical, governance, and patient safety issues to be considered by the patients, clinical teams, and their service organizations. These issues are currently mitigated by patients and clinicians working together in relationships of trust.

### Strengths and Limitations

Our study findings are likely to apply to adult populations, as they do not relate specifically to the age or clinical condition of the young people, particularly as smartphone ownership among older people is rapidly increasing [10], giving them easier access to text messaging and email. We included a relatively large number of case study sites for mixed-methods data collection covering a wide range of clinical conditions. Clinical teams were using widely used digital channels. The study captured the perspectives of many young people living with long-term conditions and those of a wide range of clinical team members. The generalizability of our study findings is limited by the study design, as with any empirical study of practice-initiated behavior. We may have detected only what is most obvious and may have missed more subtle issues. Our participants may have reported particularly positive or, perhaps to a lesser extent, negative experiences. We were unable to recruit young people who were not engaging with their health care provider. Interviews did not elicit explicit ethical reflection. Clinicians found it difficult to estimate their workload during the interviews, and we did not attempt to collect data about digital communication activity via their digital communication system. Some clinics were unable to provide the size of their patient list. We did not attempt to collect cost data for patients as we had no comparator group. We were able to estimate the direct costs associated with the delivery of providing digital communication with patients, but we did not have comparator data to estimate the costs incrementally. While we identified qualitatively how this communication could lead to NHS savings, we did not have accurate-enough incremental outcome data to quantify the savings or cost-effectiveness. The study was undertaken in the NHS, where costs of care are not made clear to individual patients.

### Comparison With Prior Work

The clinical teams we studied did not need convincing of the benefits of implementing timely digital clinical communication [48]. There is evidence that motivation and enthusiasm make a difference when implementing digital clinical interventions [49]. However, despite the importance of evaluating their digital access service for justifying its further development [50], none of the clinical teams had done so. Systematic reviews of intervention studies, usually focused on specific disease areas and published since we were preparing for this study, have mostly found some benefit from the use of digital channels for communication between patient and clinical teams, but some found no benefit. One review of text messaging for diabetes found no clear impact on glycemic control and self-management [51], whereas other reviews have found that telehealth improves glycemic control [52,53]. Systematic reviews on mental health found mostly positive findings [54,55]. A systematic review of telehealth to support family caregivers of people with chronic disease concluded that telehealth can positively affect care [56].

### Conclusions

Our study findings suggest how the introduction of timely access for patients to their clinical team using digital channels could be safely and ethically achieved, to improve the experience of health care and enhance self-management. First, implement the service initially with patients where there is an existing relationship of trust between patient and clinical team. Examples include patients with long-term conditions and women in the antenatal period. Second, focus on delivery to the population of patients where the service is responding to an identified need—for example, patients in transition between services (such as the young people we studied); patients in transition between treatments (eg, a person with diabetes starting insulin), or before or after treatment (eg, liver transplant); and patients in transition in their life (eg, starting university)—and monitor impact on staff workload. Third, prior to introducing the timely digital access, clinical teams need to work out how they will proactively manage safety [57] (eg, inadvertent information disclosure) and ethical issues (eg, role boundaries), and use their information governance specialists as a resource (eg, provision of training). Improvements in the technological infrastructure (eg, NHSmail 2 [16]) have solved, or will solve, some safety and ethical concerns, but others need to be addressed within the clinical team, often with simple measures such as a timetable of clinical team members’ availability within email signatures (see LYNC study Quick Reference Guides designed to support clinical team discussions on these issues) [44].

Introducing timely access for patients to their clinical team using digital channels will require trained leadership [15] and patient involvement [50].

An experimental research design is needed to evaluate the impact of timely digital access to clinical teams on health outcome and health care provision. The research will need to take account of the nature of the existing relationship between patient and clinician.

## Abbreviations

NHS: National Health Service
NIHR: National Institute for Health Research
